# Supplementary material for: Complex‐centric proteome profiling by SEC‐SWATH‐MS
Source: Mol Syst Biol. 2019 Jan 14;15(1):e8438. doi: 10.15252/msb.20188438 (PMC6346213; doi:10.15252/msb.20188438)
Supplement: Supplementary file 8 — Dataset EV7 [file MSB-15-e8438-s008.zip › feature_plots_string/Q15573.pdf]

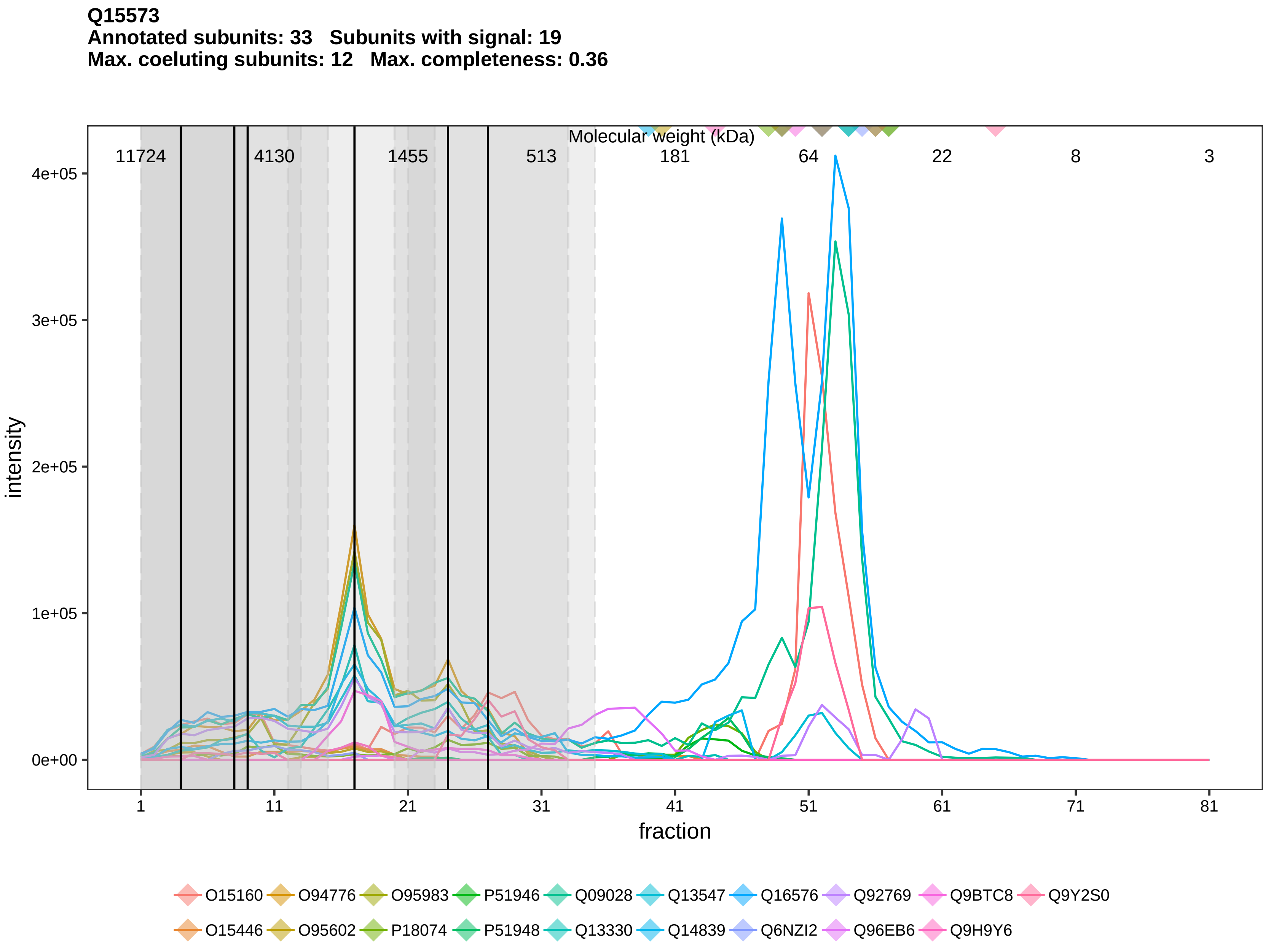
Q15573
Annotated subunits: 33 Subunits with signal: 19
Max. coeluting subunits: 12 Max. completeness: 0.36
Molecular weight (kDa)
11724
4130
1455
513
181
64
22
8
3
intensity
4e+05
3e+05
2e+05
1e+05
0e+00
fraction
1
11
21
31
41
51
61
71
81
O15160
O94776
O95983
P51946
Q09028
Q13547
Q16576
Q92769
Q9BTC8
Q9Y2S0
O15446
O95602
P18074
P51948
Q13330
Q14839
Q6NZI2
Q96EB6
Q9H9Y6
